# Supplementary material for: AI‐powered nursing handoffs: Introducing and evaluating the patient report template
Source: Learn Health Syst. 2025 Jul 10;10(Suppl 1):e70026. doi: 10.1002/lrh2.70026 (PMC13239256; doi:10.1002/lrh2.70026)
Supplement: Supplementary file 1 — DATA S1. Supporting Information. [file LRH2-10-e70026-s001.docx]

Table A1. Patient Profile Section and Example Data

| **Component** | **Example Data** |
| --- | --- |
| Name | *Jane Doe* |
| Age | *60* |
| Sex | *Female* |
| Physician | *Dr. John Smith, M.D.* |
| Identifiers | *MRN: 0000-00-0000*  *DOB: 01/01/1964* |
| Location | *Neurology Unit, Room 123* |
| Code Status | *DNR* |
| Isolation Status | *None* |

Table A2. Review of Systems Section and Example Data

| **Component** | **Example Data** |
| --- | --- |
| Chief Complaint | *Seizure* |
| Constitutional | *Emergency Severity Index (ESI) Level 2, indicating high risk situations or vital sign instability* |
| Eyes | *Diplopia, unspecified glaucoma, heteronymous bilateral field defects* |
| ENT | *Other speech disturbances* |
| Cardiovascular | *No specific cardiovascular complaints noted* |
| Respiratory | *Obstructive sleep apnea* |
| Gastrointestinal | *No specific GI complaints noted* |
| Genitourinary | *No specific GU complaints noted* |
| Musculoskeletal | *Weakness* |
| Integumentary | *No specific dermatologic complaints noted* |
| Neurological | *Unspecified convulsions, benign intracranial hypertension, headache, congenital hydrocephalus, presence of cerebrospinal fluid drainage device* |
| Psychiatric | *Major depressive disorder, single episode* |
| Endocrine | *Obesity, unspecified* |
| Hematologic / Lymphatic | *No specific hematologic/lymphatic complaints noted* |
| Allergic / Immunologic | *Allergies noted to Carbamazepine Derivatives, Phenytoin, Codeine, Depakote, Benadryl Decongestant, Iodine, Influenza Virus Vaccine, Dilaudid (PF), morphine, prednisone, Tdap vaccine* |

Table A3. Situation Section and Example Data

| **Component** | **Example Data** |
| --- | --- |
| Level of Uncertainty | *Given the multiple diagnoses, differential diagnosis may include various neurological disorders* |
| Recent Changes | *Patient admitted for seizure activity* |
| Response to treatments | *Responding to treatments as evidenced by transferred from Emergency Department to Neurology* |
| Plan of care | *Monitoring in Neurology, medication administration as per physician's orders* |

Table A4. Safety Section and Example Data

| **Component** | **Example Data** |
| --- | --- |
| **Lab Values** | *Bicarbonate: 21.0 (low: 22.0, high: 32.0) [abnormal]*  *MCHC: 30.2 (low: 32.0, high: 37.0) [abnormal]*  *Glucose: 128.0 (low: 70.0, high: 100.0) [abnormal]*  *RDW-SD: 50.8 (low: 35.1, high: 46.3) [abnormal]* |
| **Allergies** | *Carbamazepine Derivatives, Phenytoin, Codeine, Depakote, Benadryl Decongestant, Iodine, Influenza Virus Vaccine, Dilaudid (PF), morphine, prednisone, Tdap vaccine* |
| **Alerts** | *None specified* |

Table A5. Background Section and Example Data

| **Component** | **Example Data** |
| --- | --- |
| **Comorbidities** | *Asthma, chronic stable*  *Pruritus*  *Pseudotumor cerebri*  *Chronic headache*  *Seizures vs pseudoseizures* |
| **Previous Episodes** | *Previous hospitalizations for congenital hydrocephalus, vertigo, dizziness, and pseudoseizures*  *Previous surgeries including cochlear implants, VP shunt, LS shunt, umbilical hernia repair, laparoscopic cholecystectomy* |
| **Current Medications** | *Acetaminophen, azelastine, albuterol sulfate, montelukast, fluticasone, omeprazole, ibuprofen, acetazolamide, cholestyramine, topiramate, lorazepam, sodium chloride flush* |
| **Family History** | *Neurology note reports seizures in mother after head trauma* |

Table A6. Actions Section and Example Data

| **Component** | **Example Data** |
| --- | --- |
| **Actions Taken** | *Admitted to Neurology*  *Physician's orders include radiology (CT Scan, General Xray), lab tests, IV therapy, medications, general care monitoring, and therapeutic devices* |
| **Actions Required / Ongoing** | *Continued monitoring and medication administration as per physician's orders*  *Further evaluation by Neurology and Neurosurgery as required* |
| **Rationale** | *Monitoring and medication to manage and mitigate seizure activity and associated symptoms* |
| **Signs to elevate** | *Increased seizure activity, changes in neurological status, severe headache, dizziness* |

Table A7. Timing Section and Example Data

| **Component** | **Example Data** |
| --- | --- |
| **Levels of Urgency / Prioritization** | *Monitoring for seizure activity*  *Medication administration*  *Further diagnostic imaging if required* |
| **Explicit Timing** | *None specified* |
| **Coordination** | *Coordination with Neurology, Neurosurgery, and Radiology* |

Table A8. Ownership Section and Example Data

| **Component** | **Example Data** |
| --- | --- |
| **Responsibility** | *Neurology team in charge of care* |
| **Patient/Family** | *Medical decisions to be made by patient or designated family member* |

Table A9. Next Section and Example Data

| **Component** | **Example Data** |
| --- | --- |
| **Anticipated Changes** | *Possible changes in neurological status depending on response to interventions* |
| **Plan of action** | *Continued monitoring and medication administration* |
| **Contingency Plans** | *If seizure activity increases, escalate care to more intensive monitoring or intervention*  *If neurological status changes, re-evaluate treatment plan* |

Table A10. Nurses’ ratings of the Patient Profile section

| **Patient Profile** | **Mode** | **Median** | **Average** | **Minimum** | **Maximum** | **Count** |
| --- | --- | --- | --- | --- | --- | --- |
| Name: Patient's Name | 5 | 5 | 4.42 | 1 | 5 | 106 |
| Age: Patient's age | 5 | 5 | 4.25 | 1 | 5 | 106 |
| Sex: Biological Sex of the patient | 5 | 4 | 4.03 | 1 | 5 | 105 |
| Physician: Attending physician | 5 | 4 | 4.16 | 1 | 5 | 106 |
| Identifiers: e.g., Medical Record Number (MRN), date of birth (DOB) | 5 | 5 | 4.34 | 1 | 5 | 105 |
| Location: Location within the healthcare facility (e.g., room number, unit) | 5 | 4 | 4.20 | 1 | 5 | 106 |
| Code Status: e.g., DNR | 5 | 5 | 4.38 | 1 | 5 | 106 |
| Isolation Status: e.g., Contact isolation, droplet isolation | 5 | 5 | 4.28 | 1 | 5 | 106 |

Table A11. Nurses’ ratings of the Review of Systems section

| **Review of Systems** | **Mode** | **Median** | **Average** | **Minimum** | **Maximum** | **Count** |
| --- | --- | --- | --- | --- | --- | --- |
| Chief Complaint: The primary issue or symptoms that brought the patient into care or the main concern for the current admission | 5 | 5 | 4.4 | 1 | 5 | 106 |
| Integumentary: Dermatologic conditions, breast cancer, Rashes, skin changes, breast lumps | 5 | 4 | 3.88 | 1 | 5 | 106 |
| Neurological: Neurological disorders (e.g., stroke, epilepsy), Headaches, seizures, numbness | 5 | 4 | 4.14 | 1 | 5 | 106 |
| Psychiatric: Mental health conditions, Depression, anxiety, sleep issues | 5 | 4 | 3.98 | 1 | 5 | 106 |
| Endocrine: Endocrine disorders (e.g., diabetes, thyroid), Polyuria, weight changes, intolerance | 5 | 4 | 3.99 | 1 | 5 | 106 |
| Hematologic/Lymphatic: Blood disorders (e.g., anemia, clotting issues), Bruising, bleeding, lymphadenopathy | 5 | 4 | 4.08 | 1 | 5 | 106 |
| Allergic/Immunologic: Allergies, immunodeficiencies, autoimmune diseases, Allergies, recurrent infections | 5 | 5 | 4.18 | 1 | 5 | 106 |
| Constitutional: General health overview, systemic issues, Fever, weight loss, fatigue, vitals | 5 | 5 | 4.12 | 1 | 5 | 106 |
| Eyes: Ocular diseases, systemic conditions, Vision changes, pain, redness | 5 | 4 | 3.73 | 1 | 5 | 106 |
| ENT: ENT conditions, infections, allergies, Hearing loss, congestion, sore throat | 5 | 4 | 3.85 | 1 | 5 | 106 |
| Cardiovascular: Heart diseases (e.g., MI, heart failure), Chest pain, palpitations, dyspnea | 5 | 5 | 4.23 | 1 | 5 | 106 |
| Respiratory: Respiratory conditions (e.g., asthma, pneumonia), Cough, shortness of breath, wheezing | 5 | 5 | 4.24 | 1 | 5 | 106 |
| Gastrointestinal: GI disorders (e.g., infections, IBD), Nausea, vomiting, abdominal pain | 5 | 4 | 3.93 | 1 | 5 | 106 |
| Genitourinary: UTIs, renal conditions, reproductive health, Dysuria, hematuria, pelvic pain | 5 | 4 | 3.89 | 1 | 5 | 106 |
| Musculoskeletal: Musculoskeletal disorders (e.g., arthritis), Joint pain, muscle weakness | 5 | 4 | 3.87 | 1 | 5 | 106 |

Table A12. Nurses’ ratings of the Situation Section

| **Situation** | **Mode** | **Median** | **Average** | **Minimum** | **Maximum** | **Count** |
| --- | --- | --- | --- | --- | --- | --- |
| Level of Uncertainty: Attempt to quantify uncertainty, differential diagnosis, etc. | 4 | 4 | 3.25 | 1 | 5 | 106 |
| Recent Changes: Any recent changes to patient's condition | 5 | 5 | 3.96 | 1 | 5 | 106 |
| Response to treatments: How is the patient responding to given interventions | 5 | 5 | 3.88 | 1 | 5 | 106 |
| Plan of care: Current nursing plan of care | 4 & 5 | 4 | 3.95 | 1 | 5 | 105 |

Table A13. Nurses’ ratings of the Safety section

| **Safety** | **Mode** | **Median** | **Average** | **Minimum** | **Maximum** | **Count** |
| --- | --- | --- | --- | --- | --- | --- |
| Lab Values: Results of labs for the patient, anomalous labs first, routine results separate, chronological from most recent | 5 | 4 | 4.21 | 1 | 5 | 106 |
| Allergies: Any medication, food, or other allergies relevant to care | 5 | 5 | 4.36 | 1 | 5 | 106 |
| Alerts: fall, isolation, etc. | 5 | 4 | 4.22 | 1 | 5 | 106 |

Table A14. Nurses’ ratings of the Background Section

| **Background** | **Mode** | **Median** | **Average** | **Minimum** | **Maximum** | **Count** |
| --- | --- | --- | --- | --- | --- | --- |
| Comorbidities: Other diseases such as diabetes, coronary, etc. | 4 | 4 | 4.11 | 1 | 5 | 106 |
| Previous Episodes: Previous hospitalizations, surgeries, illnesses, complications | 4 & 5 | 4 | 3.91 | 1 | 5 | 106 |
| Current Medications: Current list of medications used by patients and relevant medications ceased | 5 | 5 | 4.29 | 1 | 5 | 106 |
| Family History: e.g., Hereditary conditions, history of chronic disease | 3 & 4 | 3 | 3.36 | 1 | 5 | 106 |

Table A15. Nurses’ Ratings of the Actions section

| **Actions** | **Mode** | **Median** | **Average** | **Minimum** | **Maximum** | **Count** |
| --- | --- | --- | --- | --- | --- | --- |
| Actions Taken: Actions and interventions that have already been completed | 4 | 4 | 4.03 | 1 | 5 | 106 |
| Actions required/ongoing: Explain any treatments or procedures currently in progress or that need to be completed still | 5 | 4 | 4.08 | 1 | 5 | 106 |
| Rationale: Justification for previous and current/future actions | 4 & 5 | 4 | 3.71 | 1 | 5 | 105 |
| Signs to elevate: Symptoms which indicate need to alert physician/nurse practitioner | 5 | 4 | 4.17 | 1 | 5 | 105 |

Table A16. Nurses’ Ratings of the Timing section

| **Timing** | **Mode** | **Median** | **Average** | **Minimum** | **Maximum** | **Count** |
| --- | --- | --- | --- | --- | --- | --- |
| Levels of Urgency/ Prioritization: Order of actions of care in decreasing priority | 5 | 4 | 3.88 | 1 | 5 | 106 |
| Explicit Timing: If specific timestamps for certain actions (administer medication, remove ventilator) | 5 | 4 | 3.94 | 1 | 5 | 106 |
| Coordination: Mention any coordination needed with other departments or services | 4 | 4 | 3.72 | 1 | 5 | 106 |

Table A17. Nurses’ ratings of the Ownership section

| **Ownership** | **Mode** | **Median** | **Average** | **Minimum** | **Maximum** | **Count** |
| --- | --- | --- | --- | --- | --- | --- |
| Responsibility: What person/team in charge of care | 5 | 4 | 4.19 | 1 | 5 | 105 |
| Patient/Family: Who is making medical decisions for patient | 5 | 4 | 3.95 | 1 | 5 | 105 |

Table A18. Nurses’ ratings of the Next section

| **Next** | **Mode** | **Median** | **Average** | **Minimum** | **Maximum** | **Count** |
| --- | --- | --- | --- | --- | --- | --- |
| Anticipated Changes: Any possible/likely changes in status for patients because of interventions/care | 4 | 4 | 3.8 | 1 | 5 | 106 |
| Plan of action: If current conditions persist, plan of care for patient | 5 | 4 | 3.98 | 1 | 5 | 106 |
| Contingency Plans: If current conditions change in specific ways, adaptations to plan or other plans of care for the patient | 4 | 4 | 3.95 | 1 | 5 | 106 |

Table A19. Nurses’ ratings for Perceptions of AI

| **Perceptions of AI** | **Mode** | **Median** | **Average** | **Minimum** | **Maximum** | **Count** |
| --- | --- | --- | --- | --- | --- | --- |
| Comfort using AI in your work (1-5) | 2 | 2 | 2.38 | 1 | 4 | 105 |
| Level of Trust (1-5) | 2 | 2 | 2.5 | 1 | 5 | 105 |
| Expected Utility (1-5) | 3 | 3 | 3 | 1 | 5 | 106 |

Table A20. Composition by Unit of sampled Nurse Population

| **Department** | **Percent** | **Count** |
| --- | --- | --- |
| Cardiothoracic Surgery | 2.94 | 3 |
| Emergency Medicine | 2.94 | 3 |
| Family Medicine | 1.96 | 2 |
| Internal Medicine | 8.82 | 9 |
| Microbiology and Immunology | 0.98 | 1 |
| Neurology | 4.90 | 5 |
| Neurosurgery | 2.94 | 3 |
| Obstetrics and Gynecology | 9.80 | 10 |
| Ophthalmology and Visual Sciences | 1.96 | 2 |
| Orthopedics and Rehabilitation | 0.98 | 1 |
| Otolaryngology—Head and Neck Surgery | 0.98 | 1 |
| Pathology | 0.98 | 1 |
| Pediatrics, Stead Family Department of Pediatrics | 17.65 | 18 |
| Psychiatry | 2.94 | 3 |
| Radiation Oncology | 1.96 | 2 |
| Radiology | 1.96 | 2 |
| Surgery | 3.92 | 4 |
| Urology | 2.94 | 3 |
| Hospital Dentistry | 0.98 | 1 |
| Other | 27.45 | 28 |

Table A21. Composition by years of experience of sampled nurse population

| **Years of Experience** | **Percent** | **Count** |
| --- | --- | --- |
| 0-10 Years | 53.77 | 57 |
| 11-20 Years | 21.70 | 23 |
| 21-30 Years | 24.53 | 26 |
